# Understanding the Plasmonic Effect of Enhanced Photodegradation with Au Nanoparticle Decorated ZnO Nanosheet Arrays under Visible Light Irradiation

**DOI:** 10.3390/molecules28196827

**Published:** 2023-09-27

**Authors:** Jun Wang, Dongliang Liu, Shun Yuan, Bo Gao, Lin Cheng, Yu Zhang, Kaijia Chen, Aimin Chen, Lianbi Li

**Affiliations:** 1 School of Science, Xi’an Polytechnic University, 19 Jinhua South Road, Xi’an 710048, China; l295936493@126.com (D.L.); shunyuan_china@163.com (S.Y.); g17691005744@163.com (B.G.); chenglin@xpu.edu.cn (L.C.); 13091138761@163.com (K.C.); chenaimin_xa@163.com (A.C.); xpu_lilianbi@163.com (L.L.); 2 Engineering Research Center of Flexible Radiation Protection Technology, Xi’an Polytechnic University, 19 Jinhua South Road, Xi’an 710048, China; 3 School of Science, Xi’an Jiaotong University, 28 Xianning Road, Xi’an 710049, China; zhangyu.fengyun3@stu.xjtu.edu.cn

**Keywords:** Au nanoparticle decorated ZnO nanosheets arrays, visible light plasmon-enhanced photocatalysis, hot electron injection, electromagnetic simulations

## Abstract

Plasmonic-enhanced photocatalysis using visible light is considered a promising strategy for pollution photodegradation. However, there is still a lack of comprehensive and quantitative understanding of the underlying mechanisms and interactions involved. In this study, we employed a two-step process to fabricate arrays of ZnO nanosheets decorated with Au nanoparticles (Au-ZnO NS). Various characterization techniques were used to examine the morphological, structural, and chemical properties of the fabricated Au-ZnO NS array. Furthermore, we systematically investigated the photocatalytic degradation of methyl orange under visible light irradiation using Au-ZnO NS arrays prepared with varying numbers of photochemical reduction cycles. The results indicated that as the number of photochemical reduction cycles increased, the photodegradation efficiency initially increased but subsequently decreased. Under visible light irradiation, the Au-ZnO NS array obtained via four cycles of photochemical reduction exhibits the highest photocatalytic degradation rate of methyl orange 0.00926 min^−1^, which is six times higher than that of the ZnO NS array. To gain a better understanding of the plasmonic effect on photodegradation performance, we utilized electromagnetic simulations to quantitatively investigate the enhancement of electric fields in the Au-ZnO NS array. The simulations clearly presented the nonlinear dependencies of electric field intensity on the distribution of Au nanoparticles and the wavelength of radiation light, leading to a nonlinear enhancement of hot electron injection and eventual plasmonic photodegradation. The simulated model, corresponding to four cycles of photochemical reduction, exhibits the highest electric field intensity at 550 nm, which can be attributed to its strong plasmonic effect. This work provides mechanistic insights into plasmonic photocatalysts for utilizing visible light and represents a promising strategy for the rational design of high-performance visible light photocatalysts.

## 1. Introduction

Zinc oxide (ZnO) has recently gained attention in the field of photocatalytic degradation due to its high chemical stability, low cost, and non-toxicity [1]. However, its inherent bandgap limitations present a challenge in utilizing the full spectrum of solar irradiation [2]. ZnO can only absorb light below 380 nm, thereby restricting its utilization of visible light. To address this limitation, researchers have explored various strategies to enhance the light absorption and photocatalytic efficiency of ZnO in the visible light region [3], including metallic ion doping [4,5], non-metal doping [6], noble metal deposition [7,8], and semiconductor coupling [9,10]. Among these strategies, the modification of ZnO nanostructures with Au nanoparticles (Au NPs) has gained significant attention from researchers in recent years due to its versatile synthesis methods and exceptional properties [11]. Au NPs possess unique localized surface plasmonic resonance (LSPR) properties, enabling an efficient absorption and localization of light in the visible regions [12]. Through the plasmonic effect of Au NP-decorated ZnO nanostructures, not only does the light absorption range expand, but also the separation and utilization of photogenerated charge carriers improve, resulting in enhanced photocatalytic performance.

Considerable effort has been devoted to fabricating Au NP-decorated ZnO nanostructures (Au-ZnO) using various methods, as reported in the literature [11]. Within the realm of Au-ZnO NPs, Rehman et al. [13] reported the phytogenic fabrication of Au-ZnO NPs employing aqueous leaf extracts of pecan nuts as a reducing agent. Ahmed et al. [14] developed a synthesis method for Au NPs homogeneously distributed on hexagonal ZnO NPs using polyvinylpyrrolidone (PVP) as a pore and structure-directing agent. Waclawik et al. [15] investigated Au deposition on large ZnO nanoparticles by controlling the rate of photoreduction of metastable AuCl^2–^ at the solid–solution interface. Regarding Au-ZnO arrays, Kuriakose et al. [16] proposed the formation of Au-ZnO nanohybrids via the sputter deposition of an Au thin film onto a ZnO film, followed by annealing. Hu et al. [17] utilized sodium citrate as a reducing agent to fabricate ultrathin two-dimensional ZnO nanosheets decorated with Au NPs. Kang et al. [18] described the synthesis of plasmonic Au-sensitized ZnO nanowire arrays using chemical vapor deposition and subsequent photoreduction techniques.

While the fabrication of Au NPs decorated ZnO nanostructures has received significant attention, limited focus has been placed on understanding the mechanism of plasmonic photocatalysis [19,20,21,22,23,24]. Zhang et al. [19] conducted a systematic investigation of the fundamental physical mechanisms of plasmonic photocatalysis, highlighting the presence of a Schottky junction and LSPR properties. Leong et al. [20] clarified the mechanism of LSPR for various noble metal nanoparticles, as well as the Schottky phenomenon on the metal-semiconductor photocatalysts studied. Bora et al. [21] compared possible electron transfer mechanisms using plasmonic Ag-ZnO NRs and Au-ZnO NRs under both UV and visible light excitations. Wu et al. [22] illustrated charge transfer in Au NP–nonpolar ZnO photocatalysts via a surface-potential-derived three-dimensional band diagram. Bruno et al. [23] investigated localized energy band bending in ZnO Nanorods decorated with Au NPs. Zhou et al. [24] analyzed the LSPR effect on the photodegradation of Au nanorods-ZnO microspheres driven by visible light irradiation. Nevertheless, a comprehensive and quantitative understanding of the mechanisms underlying visible light plasmonic photocatalysis and the involved interactions is still lacking. The complex interplay between ZnO, Au NPs, and the surrounding environment necessitates quantitative investigation. Consequently, elucidating these mechanisms is crucial to optimize the design and performance of metal ZnO nanostructures for visible light-driven photocatalytic applications.

In this study, Au-ZnO NS arrays were prepared using a hydrothermal annealing and photochemical reduction method. By varying the numbers of Au deposition cycles, Au-ZnO NS arrays with various distributions of Au NPs were obtained and then utilized to photodegrade methyl orange (MO) and Rhodamine b (RhB). The visible light plasmonic effect of enhanced photodegradation with Au-ZnO NS arrays was experimentally and theoretically investigated via morphology analysis, absorption analysis, and electromagnetic simulations. The nonlinear dependencies of electric field intensity along the interface between Au NPs and ZnO NS on the distribution of Au NPs and the wavelength of radiation light were quantitatively discussed, which may lead to new opportunities in the visible light plasmon-enhanced photocatalytic device applications.

## 2. Results and Discussion

### 2.1. Morphological, Structural, and Chemical Characterization of Au-ZnO NS Arrays

**Figure 1:**
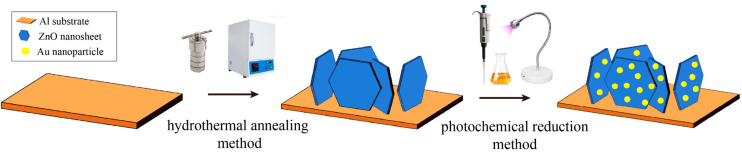
Schematic diagram of the preparation process of Au-ZnO NS array.

The fabrication strategy of the Au-ZnO NS arrays is shown schematically in Figure 1. Au-ZnO NS arrays were prepared using hydrothermal annealing and photochemical reduction methods. The details are illustrated in Section 3.2. It should be noted that the photochemical reductions were repeated at different numbers of deposition cycles (2, 4, 6, 8, and 10 cycles) to achieve Au-ZnO NS arrays with varying amounts of Au NPs. For convenience, these samples are denoted E-02, E-04, E-06, E-08, and E-10, respectively.

**Figure 2:**
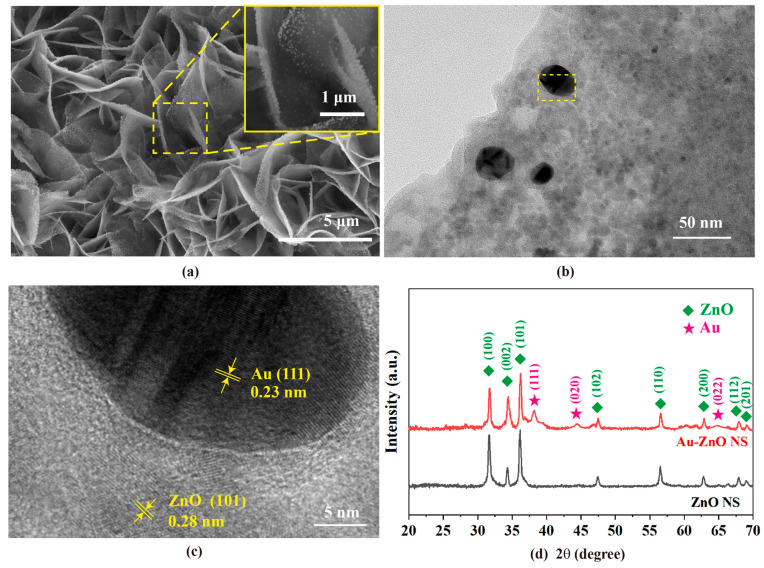
Morphological and structural characterization of typical Au-ZnO NS arrays (E-04). (**a**) SEM images. The inset is the high magnification SEM image, (**b**) TEM image (The yellow box corresponds to the region shown in (**c**), (**c**) HRTEM image, and (**d**) XRD spectra of ZnO NS and Au-ZnO NS.

The morphologies and microstructures of a typical Au-ZnO NS array were observed via scanning electron microscopy (SEM), transmission electron microscope (TEM), and X-ray diffraction analysis (XRD). Figure 2a shows the typical SEM images of Au-ZnO NS arrays (E-04). ZnO NS are covered with dense and discrete Au NPs. It can be seen that from the SEM images at high magnification in the inset of Figure 2a, the shapes of most Au NPs are quasi-spherical. Figure 2b shows the TEM image of Au-ZnO NS, from which Au NPs, having a size of 20–40 nm, are successfully decorated on the surface of the ZnO NS. From the high-resolution transmission electron microscope (HRTEM) image of Au-ZnO NS in Figure 2c, the lattice spacings are found to be 0.28 nm and 0.23 nm, corresponding to ZnO (101) and Au (111), respectively. The structural evolution of ZnO NS and Au-ZnO NS has also been examined from the XRD spectra, as shown in Figure 2d. The eight peaks of ZnO NS at 2θ of 31.67°, 34.33°, 36.16°, 47.44°, 56.54°, 66.38°, 67.91°, and 69.06° can be indexed to the (100), (002), (101), (102), (110), (200), (201), and (110) crystal planes of hexagonal wurtzite structure ZnO (COD card no. 96-900-4182). However, for Au-ZnO NS, besides the above XRD peak features of ZnO NS inherited, three additional peaks positioned at 38.18°, 44.40°, and 64.67° emerge and can be assigned to the (111), (020), and (022) crystal plane of face-centered-cubic Au (COD card no. 96-900-8464). The XRD result is in agreement with the HRTEM measurements and clearly confirms that Au NPs are successfully decorated on the ZnO NS.

**Figure 3:**
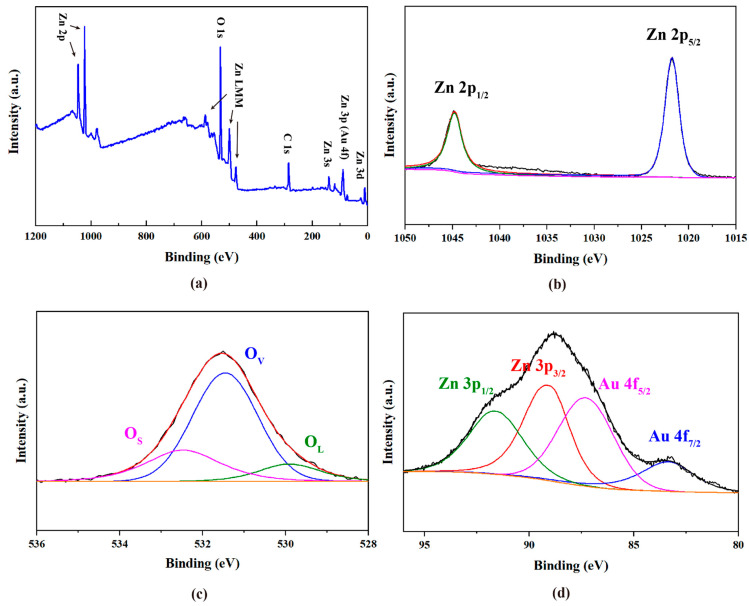
XPS analysis of typical Au-ZnO NS (E-04): (**a**) survey spectra and (**b**–**d**) narrow scan corresponding to Zn 2p, O 1s, Zn 3p, and Au 4f, respectively. Green and blue lines correspond to the fitted peaks of Zn 2p1/2, Zn 2p5/2 in (**b**). Purple, green and blue lines correspond to the fitted peaks of O_S_, O_V_ and O_L_.

In order to further clarify the existence of metallic Au NPs in the sample, X-ray photoelectron spectroscopy (XPS) analysis of typical Au-ZnO NS (E-04) was carried out, and the results are shown in Figure 3. The survey XPS spectrum in Figure 3a reveals the existence of Zn, O, and Au elements in the sample. To provide more details of composition and elemental states, the narrow scans of XPS peaks corresponding to Zn 2p, O 1s, Zn 3p, and Au 4f were analyzed via peak fitting and separation. The narrow scan of Zn 2p in Figure 3b shows sharp peaks at 1044.7 and 1021.5 eV due to Zn 2p_1/2_ and 2p_3/2_, respectively, which indicates the Zn^2+^ oxidation state in ZnO wurzite lattice [25]. For the narrow scan of O 1s in Figure 3c, three fitted peaks at 532.3 eV (O_S_), 531.3 eV (O_V_), and 529.7 eV (O_L_) are found and are related to oxygen adsorption and loosely bound oxygen (OH), oxygen vacancies in Zn-O bonds, and the lattice oxygen in the ZnO wurzite, respectively [26]. Furthermore, as shown in Figure 3d, the Au 4f peak partially overlaps with the Zn 3p peak. The binding energies of 83.3 eV, 86.9 eV, 88.4 eV, and 91.2 eV correspond to Au 4f _7/2_, Au 4f_5/2_, Zn 3p_3/2_, and Zn 3p_1/2_, respectively [27]. The binding energy of Au 4f is found to be shifted towards lower values compared to pure gold (Au^0^4f_7/2_, 84.00 eV and Au^0^4f_5/2_, 87.71 eV), which indicates that the electron transfer between ZnO and Au. In addition, the energy-dispersive X-ray spectroscopy (EDS) mapping analysis of Au-ZnO NS arrays is depicted in Figure S1. The results indicate the coexistence of Zn, O, and Au elements in Au-ZnO NS arrays.

To characterize the surface adsorption characteristics of as-fabricated samples, the surface areas of pure ZnO NS arrays and typical Au-ZnO NS arrays (including the aluminum substrates) were evaluated using a multipoint BET method. The corresponding N_2_ adsorption/desorption isotherm is shown in Figure S1. The specific surface area of the pure ZnO NS array is 20.748 m^2^/g, while the specific surface area of the Au-ZnO NS arrays is 21.545 m^2^/g. This result indicates the specific surface area of the ZnO NS array slightly increases after being modified with Au NPs.

### 2.2. Photodegradation Properties of Au-ZnO NS Arrays

**Figure 4:**
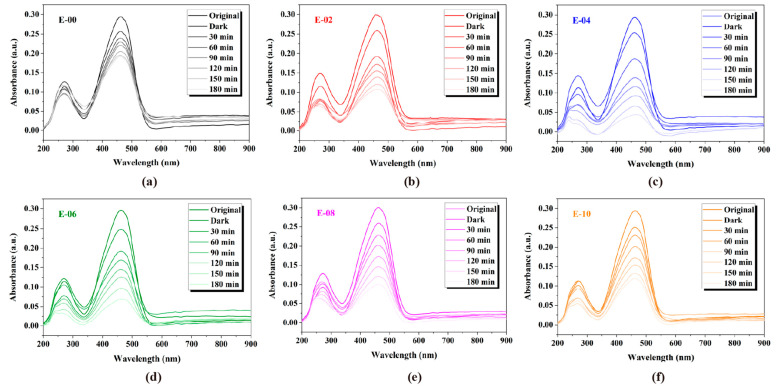
The changes of UV–Vis absorption spectrum of photodegraded MO solution as a function of irradiation time with Au-ZnO NS arrays that were prepared using various numbers of Au deposition cycles: (**a**) E-00 (pure ZnO NS array), (**b**) E-02, (**c**) E-04, (**d**) E-06, (**e**) E-08, and (**f**) E-10.

MO is a typical anionic organic dye and is widely utilized as a coloring agent in plastic, cosmetics, and food industries. Therefore, MO was adopted as a target in the photodegradation experiment. To demonstrate the photodegradation activity of Au-ZnO NS arrays, the photodegradation of MO was performed at room temperature under visible light irradiation. The changes in the UV–Vis absorption spectrum of photodegraded MO solution as a function of irradiation time with Au-ZnO NS arrays that were prepared using various numbers of Au deposition cycles are shown in Figure 4. Due to the limit by the band gap of ZnO, the photodegradation efficiency of pure ZnO NS (denoted as E-00) under visible light is low. However, after decorating Au NPs, the photodegradation efficiency of Au-ZnO NS arrays has been significantly improved. In the meantime, it can be also clearly observed from Figure 4 that the photodegradation activities of Au-ZnO NS arrays depended strongly upon a number of Au deposition cycles.

**Figure 5:**
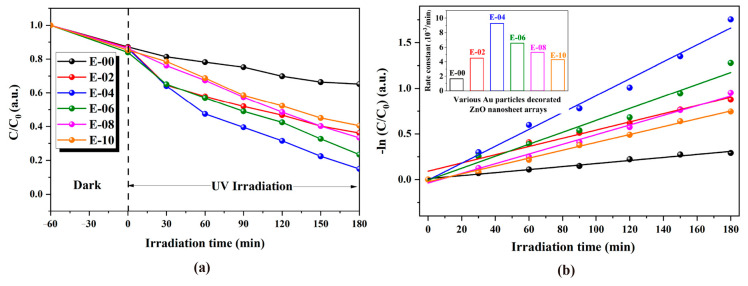
(**a**) MO concentration changes with various amounts of Au on the photocatalyst as a function of irradiation time and (**b**) pseudo-first-order kinetic fitting plots. The inset is a photodegradation rate constant comparison.

To reveal the relationship between various amounts of Au (represented as numbers of Au deposition cycles) and the reaction kinetics of the MO degradation, the photodegradation data were fitted with a pseudo-first-order kinetics equation: −ln(C/C_0_) = kt, where t, k, C, and C_0_ are the degradation time, the apparent first-order rate constant, and the MO concentrations at t and t = 0, respectively. The pseudo-first-order kinetic fitting plots of samples with various numbers of Au deposition cycles are shown in Figure 5a, and the corresponding photocatalytic rate constants are illustrated in the inset of Figure 5b. It is clearly observed that with increasing numbers of Au deposition cycles, the photodegradation rates of MO first increase and then decrease. The E-04 sample exhibited the highest photodegradation activity with a rate constant k of 0.00926 min^−1^, which is almost six times more efficient than that of pure ZnO NS arrays (0.00166 min^−1^).

The chemical oxygen demand (COD) analysis is generally used to measure the organic strength of dye pollutants and provides a viable alternative to confirm to some extent that dye is degraded rather than simply decolorized. The decrease in the COD values of MO with various Au-ZnO NS arrays is tabulated in Table S1. It was found that the highest decrease in COD values and COD removal efficiency of 92.9% belongs to E-04. This result further validates the capability of the Au-ZnO NS array to effectively degrade MO.

The degradation of RhB as an additional pollutant was also investigated to obtain a comprehensive understanding of the photodegradation ability of the prepared samples. As known, RhB is a cationic dye commonly used in papermaking, printing, and dyeing industries [28]. Comparing the degradation of MO and RhB as target pollutant molecules allows for the study of the degradation characteristics of Au-ZnO towards dye molecules with different surface electrical properties. The steps for degrading RhB were essentially the same as those for degrading MO. The photodegradation results are presented in Figure S3. The comparison of degradation rates of MO and RhB using the samples is shown in Table 1. It can be observed that as the number of Au deposition cycles increases, the degradation rates of both MO and RhB initially rise and then decline. However, the degradation effect on MO is significantly superior to that on RhB. This difference can be attributed to the surface electrical properties of the photocatalyst and target molecules. In general, the photocatalyst’s surface electrical properties are influenced by the pH value of the solution and the PZC value of the photocatalyst. If the pH of the solution exceeds the pH_PZC_, the surface of the photocatalyst carries a negative charge [29]. Conversely, if the pH of the solution is less than the pH_PZC_ [30], the surface of the photocatalyst is positively charged.

In the photodegradation experiment, the pH value of the MO solution was 6.6, and the pH value of the RhB solution was 7.5. It was reported [31] in the literature that ZnO has a point of zero charge in the pH range of 8.7–9.2. Both pH values of MO and RhB solution were lower than the PZC values of ZnO photocatalysts, indicating that the catalysts exhibited prominent electropositivity. Consequently, as MO is an anionic dye, the MO molecules are electrostatically attracted to the catalyst, resulting in a more effective catalytic effect. Conversely, since RhB is a cationic dye, the RhB molecules are electrostatically repelled by the catalyst, leading to a lower catalytic effect.

### 2.3. Plasmonic Effect of Enhanced Photodegradation under Visible Light Irradiation

**Figure 6:**
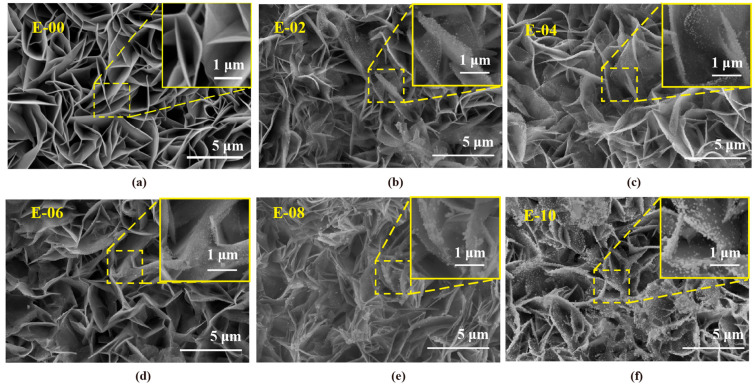
SEM images of Au-ZnO NS arrays produced with various numbers of Au deposition cycles: (**a**) E-00 (Pure ZnO NS arrays), (**b**) E-02 (2 cycles), (**c**) E-04 (4 cycles), (**d**) E-06 (6 cycles), (**e**) E-08 (8 cycles), and (**f**) E-10 (10 cycles). The insets are the high-magnification SEM images of the corresponding arrays.

In order to explain the mechanism behind the nonlinear dependence of the photodegradation performance of Au-ZnO NS arrays on the number of Au deposition cycles under visible light irradiation, surface morphology analysis of samples with various numbers of Au deposition cycles using the FE-SEM technique is represented in Figure 6. It can be observed that the amounts of Au NPs gradually increase with increasing numbers of Au deposition cycles, and the distance between Au particles becomes smaller. When the reduction reaches eight cycles, Au NPs start to aggregate, especially at the edges of ZnO NS. This may be due to the fact that photogenerated electrons will gather at the edge of ZnO NS during the photochemical reduction process [32]. More Au^3+^ at the edge will be reduced to Au^0^ and then form Au NPs. Due to space constraints, Au aggregate and even partially form discontinuous Au film.

**Figure 7:**
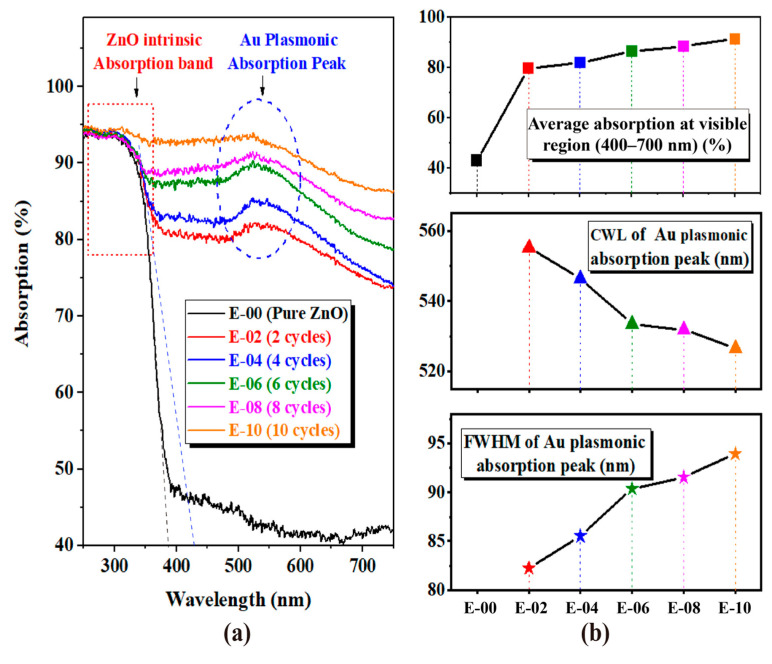
Absorption analysis of Au-ZnO NS arrays with various numbers of Au deposition cycles. (**a**) Absorption spectra and (**b**) analysis of average absorption at visible region (400–700 nm), CWL, and FWHM of absorption peak.

To further explore the plasmonic effect of Au-ZnO NS arrays, absorption analysis was conducted on samples with various numbers of Au deposition cycles placed on Al substrates using the UV-Visible Diffuse Reflectance spectrum. In order to better illustrate the influence of Au NPs on the ultraviolet absorption peak of ZnO, the original reflectance curve was replaced with an absorption spectra. Since the samples were all on opaque aluminum substrates with a transmittance of 0, the absorption spectra were calculated following the formula A = 1 − R. The absorption analysis of Au-ZnO NS arrays is presented in Figure 7, and the detailed analysis data are listed in Table S2. In Figure 7a, the intrinsic absorption bands in the UV region of pure ZnO NS can be clearly identified. With an increase in the number of Au deposition cycles, the intrinsic absorption bands are slightly broadened, as depicted in Figure 7a by the black and blue dashed lines, corresponding to samples E-00 and E-04, respectively. More importantly, the absorption in the visible region (400–700 nm) of Au-ZnO NS arrays is significantly higher than that of pure ZnO NS. As depicted in Figure 7b, the average absorption in the visible region of the as-fabricated samples is increasing from 40% for pure ZnO NS array (E-00) to more than 90% for Au-ZnO NS array (E-10). This indicates that the absorption of the as-fabricated samples in the visible spectrum has been significantly enhanced due to the modification of Au NPs. Furthermore, Figure 7a also reveals a conspicuous absorption peak in the Au-ZnO NS array occurring between 500 nm and 600 nm. By using the Gaussian Lorentz fitting technique, the center wavelength (CWL) of the absorption peak of Au-ZnO is calculated to be approximately 555 nm, with a full width at half maximum (FWHM) of about 82 nm. With an increase in the number of Au deposition cycles, a slight blue shift in the CWL of the absorption peak of Au-ZnO is observed, accompanied by a gradual broadening of the FWHM. Considering the far-field effect of the surface plasmon of metal NPs, the absorption peak can be attributed to the LSPR peak of Au NPs, and the change in its CWL and FWHM can be attributed to the plasmonic coupling effect between adjacent Au NPs [33].

The absorption analysis provides preliminary predictions and qualitative explanations for the enhanced photodegradation of samples. However, it does not fully account for the nonlinear relationship between the photodegradation efficiency and the number of Au deposition cycles for Au-ZnO NS arrays. To gain a comprehensive understanding of the visible light photocatalysis mechanism of Au-ZnO NS arrays, a quantitative analysis of the electric field enhancement effect at the Au NPs and ZnO interface is crucial.

**Figure 8:**
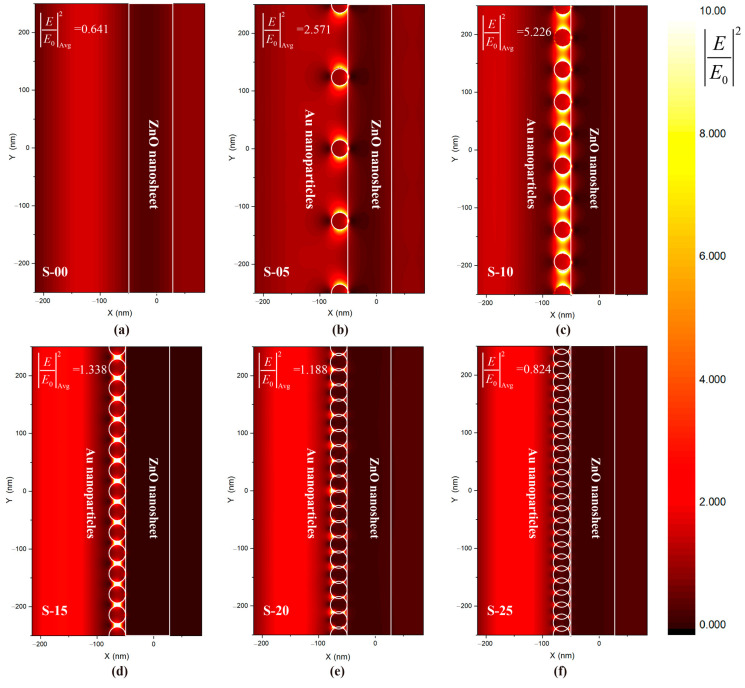
Local field intensity enhancement distribution |E/E_0_|^2^ and average local field intensity enhancement factors |E/E_0_|^2^_Avg_ along the interface of different amounts of Au NPs in 500 nanometers scales (5, 10, 15, 20 and 25) for simulated model under visible light irradiation (λ = 550 nm): (**a**) S-00, (**b**) S-05, (**c**) S-10, (**d**) S-15, (**e**) S-20, and (**f**) S-25.

Two-dimensional finite-difference time-domain (FDTD) electromagnetic simulations were used to calculate the local field intensity enhancement distribution (denoted as|E/E_0_|^2^) of pure ZnO NS and Au-ZnO NS under visible light irradiation (λ = 550 nm), where E and E_0_ represent the local and incident electric fields. The simulation results are shown in Figure 8, where varying amounts of Au NPs in scales of 500 nanometers (S-05, S-10, S-15, S-20, and S-25, representing 5, 10, 15, 20, and 25 Au NPs, respectively) are depicted. These quantities of Au NPs correspond to 2, 4, 6, 8, and 10 numbers of Au deposition cycles. It can be clearly seen that, due to the LSPR effect of Au NPs, there is a significant local field intensity enhancement near the ZnO NS in Au-ZnO NS. As the amounts of Au NPs increase, the densities of Au NPs also increase, and the distance between adjacent NPs gradually decreases. Consequently, the |E/E_0_|^2^ increases. However, when the amounts of Au NPs are too high, the Au NPs start to overlap and form a continuous film. This leads to the inability of most radiation light to incident on the surface of ZnO, resulting in a decrease in the electric field intensity of the ZnO NS.

Furthermore, the nonlinear dependence can be quantitatively observed using average local field intensity enhancement factors (denoted as |E/E_0_|^2^_Avg_) along the interface between ZnO NSs and Au NPs, as indicated in the upper left corner of Figure 8. The detailed calculation formula is given in the Supplementary Materials. As the amounts of Au NPs in the 500-nanometer scales increase, |E/E_0_|^2^_Avg_ increases from 0.641 to 5.226 and then decreases to 0.824. This corresponds to the nonlinear dependencies between photodegradation enhancement and various numbers of Au deposition cycles in the experiment.

**Figure 9:**
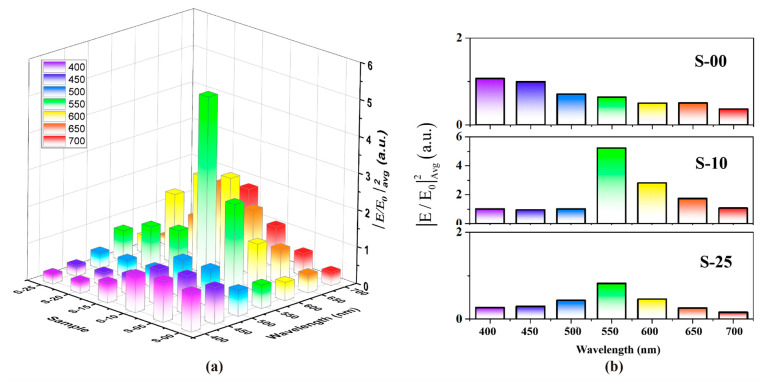
(**a**) Average local field intensity enhancement factors |E/E_0_|^2^_Avg_ under different radiation light. (**b**) Comparison of |E/E_0_|^2^_Avg_ for S-00, S-10, and S-25 under different radiation lighta.

The nonlinear dependence between the local field intensity enhancement and the irradiation wavelength also holds for Au-ZnO NS arrays. Figure 9 depicts |E/E_0_|^2^_Avg_ at the interface under different wavelengths of visible light radiation for Au-ZnO NS arrays. The detailed calculation data can be found in Table 2. It is evident from Figure 9a that |E/E_0_|^2^_Avg_ are not only influenced by the distribution of the Au NPs but also heavily dependent on the wavelength of the radiation light. When the radiation light’s wavelength is close to the LSPR resonance wavelength of the Au NPs array, the degree of localization of the electric field is higher, resulting in a more significant local field intensity enhancement effect. For instance, from the absorption analysis of the E-04 Au-ZnO NS array in Figure 7b, the LSPR resonance wavelength of the E-04 experimental sample was found to be around 550 nm. When irradiated into the S-10 simulated model via radiation light with a wavelength of 550 nm, |E/E_0_|^2^_Avg_ reaches its highest point. In contrast, pure ZnO NS arrays (denoted as S-00) have a low utilization of visible light due to the absence of the LSPR effect.

It is important to note that |E/E_0_|^2^ represents the ratio of the local field intensity to the incident field intensity rather than the actual local field intensity. The accurate computation of the actual average local field intensity (denoted as |E_actual_|^2^ _Avg_) at the interface between Au NPs and ZnO NS requires consideration of the relative spectral intensity of the radiation source. Figure S5a shows the normalized spectrum of the xenon lamp used in the degradation experiment. Due to the use of a 420–740 nm bandpass filter, the intensity of this light source at 400 nm is weak, while the light intensity near 600 nm is strong. Considering the spectral intensity at different wavelengths I_relative_, |E_actual_|^2^ _Avg_ were also calculated, as shown in Figure S5b. The detailed calculation formula of |E_actual_|^2^ _Avg_ is given in the Supplementary Materials, and the data are listed in Table S3. The |E_actual_|^2^ _Avg_ as a function of wavelength and Au nanoparticle distribution shows a similar pattern of |E/E_0_|^2^_Avg_. The S-10 sample demonstrates the strongest |E_actual_|^2^ _Avg_ at 550 nm, attributed to its robust plasmonic effect. In Table S3, |E_actual_|^2^ _Avg_ from 400–700 nm was also further calculated to confirm local field intensity enhancement under visible irradiation from the xenon lamp. Among all the samples irradiated via visible light from a xenon lamp, the S-00 sample exhibits the smallest |E_actual_|^2^ _Avg_, measuring only 0.39, while the S-10 sample demonstrates the highest |E_actual_|^2^ _Avg_, reaching up to 1.49. The |E_actual_|^2^ _Avg_ of S-10 is 3.8 times higher than that of S-00.

**Figure 10:**
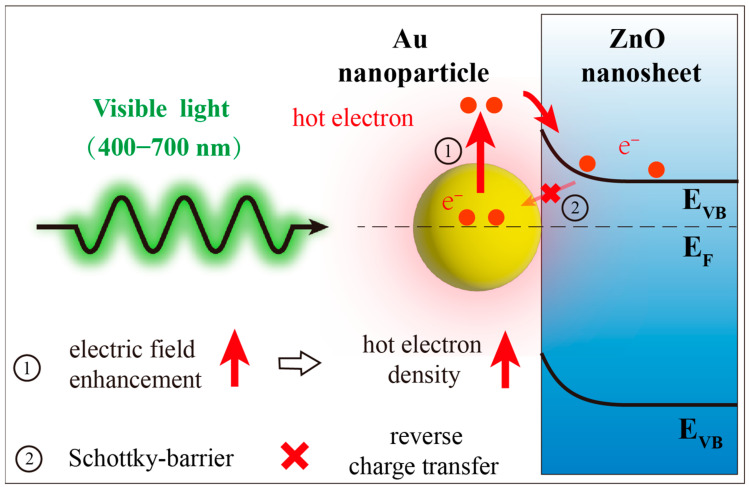
A scheme for understanding visible light plasmonic photodegradation with Au-ZnO NS arrays.

Based on the above analysis, the visible light plasmon-enhanced photocatalytic mechanism and charge transfer processes of Au-ZnO NS arrays are presented in Figure 10. Under visible light irradiation, ZnO NS cannot effectively generate electrons and holes because the energy of photons is smaller than the intrinsic bandgap width of ZnO. At this time, the electrons on the ZnO NS mainly rely on the charge transfer process. The hot electrons generated from the LSPR of Au NPs via visible light illumination are injected into the conduction band (CB) of ZnO [16]. The distribution of Au NPs and the frequency of the radiation light affect the electric field enhancement effect and the density of transferable hot electrons, ultimately influencing the visible light photodegradation efficiency in the composite structure.

In addition, the Schottky barrier formed at the interface between Au and ZnO prevents the reverse charge transfer [21]. This further enhances the utilization of charge-transferred electrons in Au-ZnO NS arrays for photodegradation.

## 3. Materials and Methods

### 3.1. Materials

Zinc nitrate hexahydrate (Zn(NO_3_)_2_·H_2_O), hexamethylenetetramine (HMTA), methyl orange (MO), and hydrogen tetrachloroaurate (III) hydrate (HAuCl_4_·3H_2_O) were procured from Shanghai Chemical Reagent Company (Shanghai, China) and Shanghai Aladdin Biochemical Technology Company Limited (Shanghai, China), respectively. Aluminum foils (0.3 mm thickness) used in this experiment were purchased from Suzhou Metal Material Company (Suzhou, China). All chemicals were used without additional purification.

### 3.2. Preparation of Au-ZnO NS Arrays

ZnO NS arrays were fabricated using a one-step hydrothermal method proposed in our previous work [34]. Subsequently, the nanosheets were annealed in air at 600 °C for 1 h (referred to as the hydrothermal annealing method). Following that, a 0.02 mol/L HAuCl_4_ solution was prepared, and 50 μL of it was applied evenly onto the 1 × 2 cm ZnO NS using a pipette gun. Then, a monochromatic LED lamp (365 nm, 8 W) was used to irradiate the sample for 2 min to reduce the Au^3+^ to Au nanoparticle, thus achieving the purpose of modifying the ZnO NS.

### 3.3. Characterization of Au-ZnO NS Arrays

The as-fabricated Au-ZnO NS arrays were characterized using various techniques. SEM images were obtained using the field-emission scanning electron microscope (FEI Quanta 450, Hillsboro, OR, USA). TEM observation was carried out using the high-resolution electron microscope (JEOL JEM-2100Plus, Tokyo, Japan). XRD spectra were measured using X-ray diffraction (BUKER D8 Advance, Karlsruhe, Germany) with Cu Kα radiation (λ = 1.5406 Å) in the 2-theta range between 20° and 70°. XPS measurements were recorded using the X-ray photoelectron spectroscopy (THERMO Escalab 250Xi, Waltham, MA, USA) equipped with a monochromatized Al-Kα X-ray source and were calibrated according to the energy of the standard C 1s peak (284.8 eV). The specific surface areas of the fabricated samples were measured using a nitrogen gas adsorption/desorption surface area tester (JWGB BK400, Beijing, China) at 77 K and were calculated using the multipoint Brunauer–Emmett–Teller (BET) method. UV-visible DRS were obtained using the fiber optic spectrometer (IDEAOPTICS Nova, Shanghai, China) equipped with an integrating sphere with assembly and 100% commercial BaSO_4_ as the reflectance sample.

In the experiment, the as-fabricated Au-ZnO NS arrays were mainly characterized in the form of powder and thin film. The Au-ZnO NS powder, which is exfoliated from the substrate, was utilized for TEM, XPS, and XRD measurements. It solely contains information about the Au particles and zinc oxide nanosheets, without substrate information. On the other hand, the Au-ZnO film refers to the actual form of the Au-ZnO NS array with the presence of a substrate. This form was used for SEM, UV-Vis DRS, and photodegradation tests.

### 3.4. Photodegradation with Au-ZnO NS Arrays

In order to characterize the photodegradation activity of Au-ZnO NS, the photodegradation of MO and RhB solution (10 mg/L) was performed at room temperature under visible light irradiation. First, five pieces of 1 × 2 cm Au-ZnO NS array were placed in a 50 mL MO solution for 1 h under dark conditions to ensure that the adsorption–desorption balance between the nanosheet arrays and the MO solution was reached. Then, the photodegradation of the pollutant solution was carried out under the irradiation of a 100 W Xenon lamp for 3 h. Every half an hour, 0.5 mL of the degraded solution was taken as samples. To generate visible light, the irradiation from the Xenon lamp is filtered using a band pass filter with a wavelength range of 420 nm to 740 nm. Finally, the adsorption spectrum of the samples was recorded using the fiber optic spectrometer (IDEAOPTICS Nova, Shanghai, China) equipped quartz cuvette to estimate the photodegradation activity of the Au-ZnO NS array. COD values of MO and RhB solutions are obtained using a COD analyzer (6B-200, Nanjing, China).

### 3.5. Electric Field Simulation of Au-ZnO NS Arrays

For a comprehensive understanding of the visible light plasmonic effect on enhanced photodegradation, 2D-FDTD electromagnetic simulations of Au-ZnO NS arrays under visible light irradiation were performed using the FDTD Solutions software (version 2020 R2) provided by Lumerical Solutions (Vancouver, Canada) [35]. These simulations were used to calculate the electric field distribution and average values along the interface between ZnO NS and Au NPs to analyze the plasmonic effect of the structure. In the simulation, the Au-ZnO NS array was described as a particle-film model, as shown in Figure S4. To characterize the optical response of samples with various numbers of Au deposition cycles, simulations were conducted for different amounts of Au NPs in 500-nanometer scales (5, 10, 15, 20, and 25), which correspond to 2, 4, 6, 8, and 10 cycles, respectively. For convenience, these simulations were named S-00, S-05, S-10, S-15, S-20, and S-25. Further details of these simulations can be found in the Supplementary Materials.

## 4. Conclusions

In this paper, Au-ZnO NS arrays were prepared via a hydrothermal method and a photochemical reduction technique. Under visible light radiation, the as-fabricated Au-ZnO NS showed a more efficient photodegradation performance of methyl orange (MO) compared to unmodified ZnO NS arrays. Interestingly, the photodegradation of MO initially increased and then decreased with increasing numbers of Au deposition cycles. The relationship between enhanced photodegradation and the number of Au deposition cycles was preliminarily explained via morphology and absorption analysis. Furthermore, the nonlinear dependences of electric field intensity on the distribution of Au NPs and the cycles of photochemical reduction were quantitatively investigated using 2D-FDTD electromagnetic simulations. Additionally, the wavelength-dependent properties of visible light photodegradation for the Au-ZnO NS array were also discussed. This study provides insights into the design of visible light plasmon-enhanced photocatalysts, opening up potential applications in various fields, such as the removal of environmental pollutants, solar water splitting, carbon dioxide photoreduction, and solar energy harvesting.

## Figures and Tables

**Table 1:** MO and RhB Photodegradation rate constant comparison with various samples.

Samples with Various Numbers of Au Deposition Cycles	E-00	E-02	E-04	E-06	E-08	E-10
MO (10^−3^/min)	1.66	4.50	9.26	6.56	5.30	4.31
RhB (10^−3^/min)	0.56	0.87	1.39	1.03	0.99	0.79

**Table 2:** |E/E_0_|^2^_Avg_ with various models under different light irradiation.

	Simulated Model	S-00	S-05	S-10	S-15	S-20	S-25
Light Irradiation Wavelength (nm)							
400		1.06986	1.12842	1.01792	0.55541	0.32871	0.26581
450		0.99581	1.05311	0.95725	0.53829	0.35629	0.29659
500		0.70619	1.01704	1.01711	0.52481	0.49423	0.43303
550		0.64138	2.57102	5.22351	1.33754	1.18838	0.82411
600		0.50409	1.28373	2.81448	2.61082	1.86501	0.46358
650		0.50702	0.93076	1.74009	2.20957	1.02707	0.25531
700		0.36422	0.60922	1.08103	1.85255	0.36574	0.15741

## Data Availability

Data are available upon reasonable request.

## Supplementary Materials

The following supporting information can be downloaded at: https://www.mdpi.com/article/10.3390/molecules28196827/s1, Figure S1: EDS mapping analysis of Au-ZnO NS arrays (a) SEM image (b) Zn mapping (c) O mapping (d) Au mapping; Figure S2: Surface areas analysis of samples (including the aluminum substrates) (a) Pure ZnO (b) Au-ZnO NS array; Table S1: COD values and removal efficiency of MO under visible light radiation with Au-ZnO NS arrays; Figure S3: The changes of UV–Vis absorption spectrum of photodegraded RhB solution as a function of irradiation time with various numbers of Au deposition cycles samples (a) E-00 (pure ZnO NS array) (b) E-02 (c) E-04 (d) E-06 (e) E-08 (f) E-10 (g) RhB concentration changes with various numbers of Au deposition cycles samples as a function of irradiation time (h) pseu-do-first-order kinetic fitting plots. The inset is a photodegradation rate constant comparison; Table S2: Analysis of average absorption at visible region, central wavelength, and FWHM of absorption peak; Figure S4: The schematic diagram of particle-film model and FDTD model details; Figure S5: (a) Spectrum of xenon lamp (adding 420–740 nm bandpass filter) (b) Actual Electric field value along the interface between ZnO NS and Au nanoparticles considering the spectral corrections of the irradiation source; Table S3: Actual electric field value along the interface with different simulated model considering the spectral corrections. Ref. [36] is cited in Supplementary Materials.

## Sample Availability

Samples are available upon reasonable request.
