# Supplementary material for: Understanding the Plasmonic Effect of Enhanced Photodegradation with Au Nanoparticle Decorated ZnO Nanosheet Arrays under Visible Light Irradiation
Source: Molecules. 2023 Sep 27;28(19):6827. doi: 10.3390/molecules28196827 (PMC10574771; doi:10.3390/molecules28196827)
Supplement: Supplementary file 1 [file molecules-28-06827-s001.zip › molecules-2605684-supplementary.pdf]

Supplementary Materials

Understanding the Plasmonic Effect of Enhanced Photodegradation with Au Nanoparticle Decorated ZnO Nanosheet Arrays under Visible Light Irradiation

Jun Wang [1,2,*], Dongliang Liu [1], Shun Yuan [1], Bo Gao [1], Lin Cheng [1,2,3], Yu Zhang [3], Kaijia Chen [1], Aimin Chen [1,2] and Lianbi Li [1,2]

[1] School of Science, Xi'an Polytechnic University, 19 Jinhua South Road, Xi'an 710048, China; l295936493@126.com (D.L.); shunyuan_china@163.com (S.Y.); g17691005744@163.com (B.G.); chenglin@xpu.edu.cn (L.C.); 13091138761@163.com (K.C.); chenaimin_xa@163.com (A.C.); xpu_lilianbi@163.com (L.L.)

[2] Engineering Research Center of Flexible Radiation Protection Technology, Xi'an Polytechnic University, 19 Jinhua South Road, Xi'an 710048, China

[3] School of Science, Xi'an Jiaotong University, 28 Xianning Road, Xi'an 710049, China; zhangyu.fengyun3@stu.xjtu.edu.cn

* Correspondence: wjunxpu@126.com

1. EDS mapping analysis of Au-ZnO NS arrays

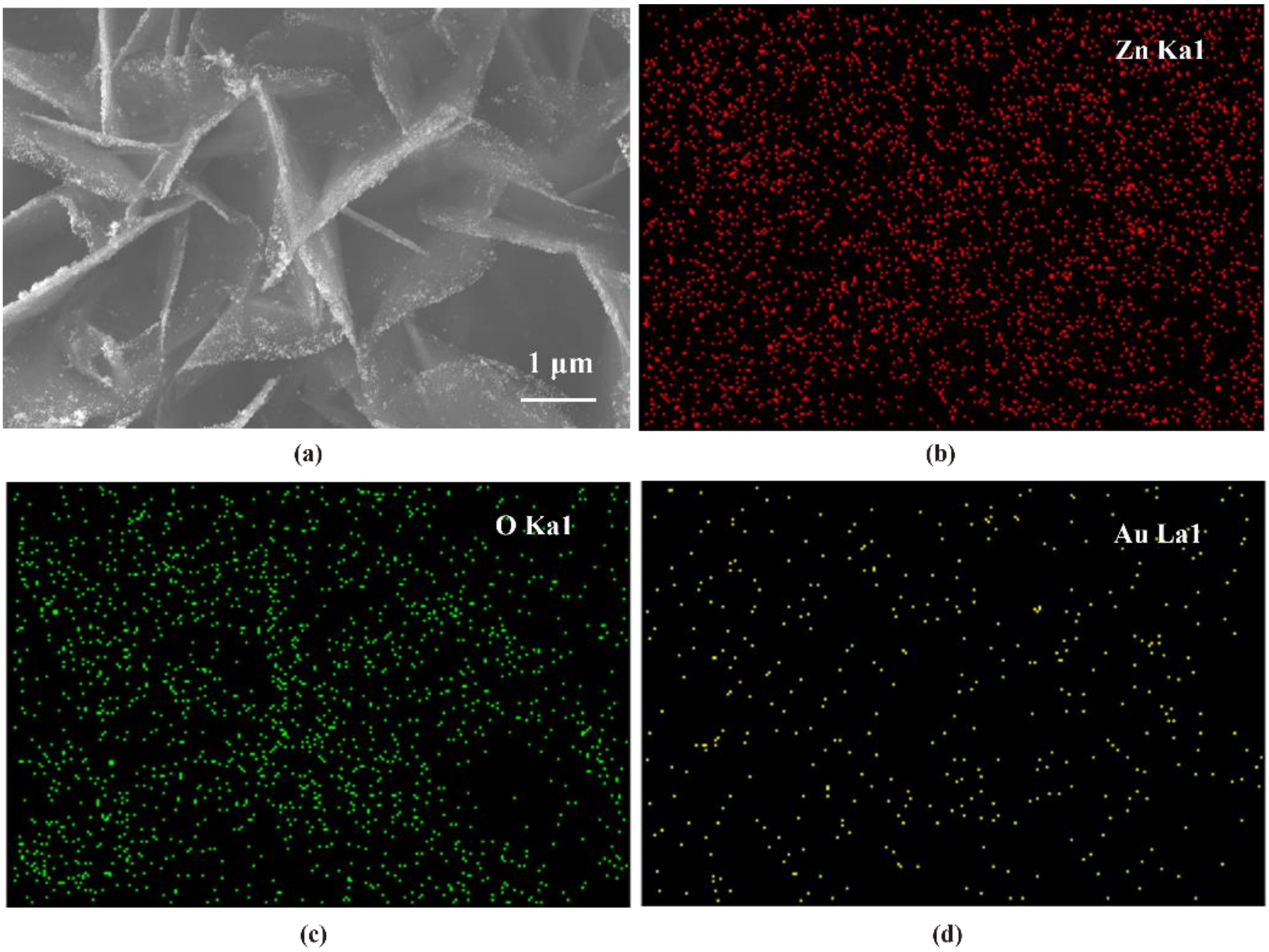

Figure S1. EDS mapping analysis of Au-ZnO NS arrays (a) SEM image (b) Zn mapping (c) O mapping (d) Au mapping

2. BET surface areas analysis (including the aluminum substrates)

The specific surface areas of pure ZnO NS and Au-ZnO NS arrays were measured using N_2 adsorption/desorption surface area tester at 77 K and were calculated by the multipoint Brunauer–Emmett–Teller (BET) method.

Because ZnO NS arrays and Au-ZnO NS arrays grown on the aluminum substrates and exhibit two-dimensional feature, the surface areas are lower than ZnO nanoparticles.

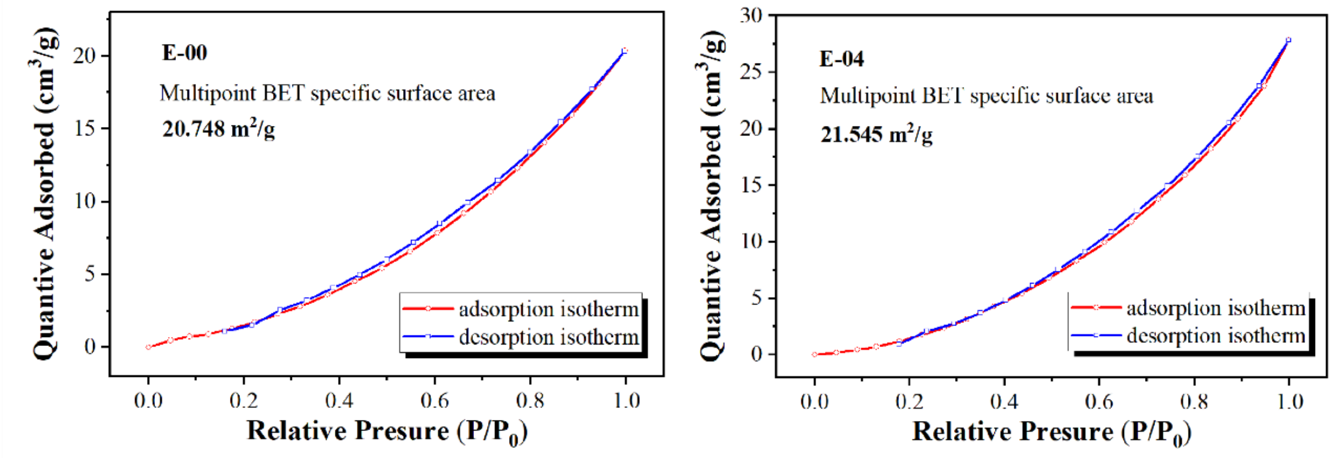

(a) (b)

Figure S2. Surface areas analysis of samples (including the aluminum substrates) (a) Pure ZnO (b) Au-ZnO NS array

3. COD analysis in the MO photodegradation

COD removal efficiency of MO is calculated from the following equation:

$$\text{COD removal efficiency} = \frac{\text{COD}_{\text{Initial}} - \text{COD}_{\text{Final}}}{\text{COD}_{\text{Initial}}} \times 100\%$$

Table S1. COD values and removal efficiency of MO under visible light radiation with Au-ZnO NS arrays

	**MO**	**E-00**	**E-02**	**E-04**	**E-06**	**E-08**	**E-10**
**COD (mg/L)**	269.183	184.271	66.651	18.932	37.837	57.141	77.414
**COD removal efficiency (%)**	——	31.5	75.2	92.9	85.9	78.7	71.2

4. RhB photodegradation with various numbers of Au deposition cycles samples

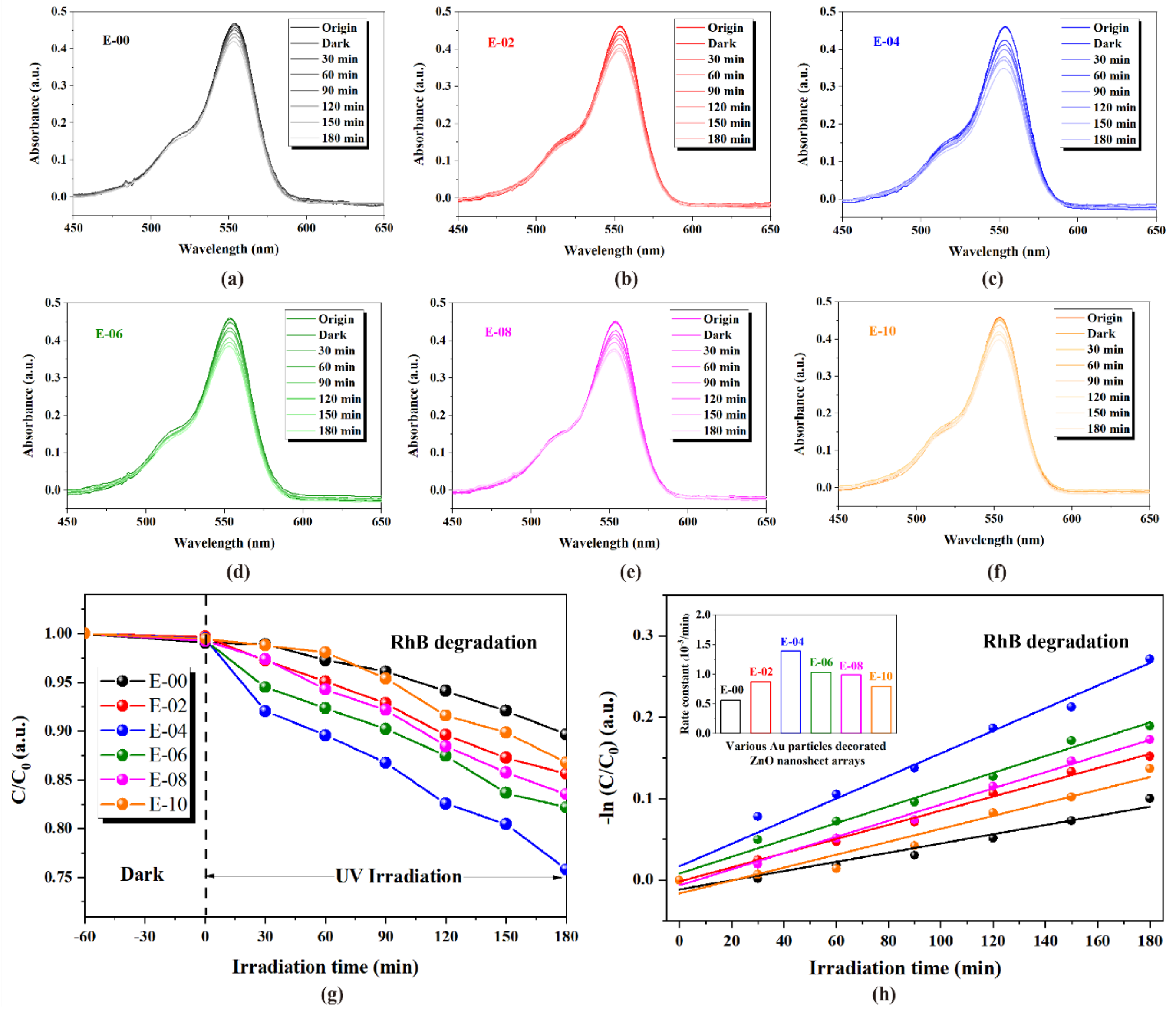

Figure S3. The changes of UV–Vis absorption spectrum of photodegraded RhB solution as a function of irradiation time with various numbers of Au deposition cycles samples (a) E-00 (pure ZnO NS array) (b) E-02 (c) E-04 (d) E-06 (e) E-08 (f) E-10 (g) RhB concentration changes with various numbers of Au deposition cycles samples as a function of irradiation time (h) pseudo-first-order kinetic fitting plots. The inset is photodegradation rate constant comparison

5. Analysis of average absorption at visible region, CWL and FWHM of absorption peak

Table S2. Analysis of average absorption at visible region, central wavelength and FWHM of absorption peak

Sample	E-00	E-02	E-04	E-06	E-08	E-10
Average absorption at visible region (%)	43.1	79.7	81.95	86.44	88.39	91.32
CWL of absorption peak (nm)	——	555.2	546.4	533.6	531.8	526.5
FWHM of absorption peak (nm)	——	82.2	85.5	90.4	91.6	93.9

6. Details of FDTD simulations

In the particle-film model, the Au NPs were of radius 15 nm and the thickness of ZnO NS is 100 nm. The model was embedding in a water environment. The dispersive dielectric constants of Au and ZnO used for simulations were taken from Palik's data [36]. The incident light is propagating perpendicular to ZnO NS and the polarization parallel to its surface. Considering the periodicity and extensibility of gold nanoparticles array, periodic boundaries conditions (PBCs) along y directions and perfectly matched layer (PML) boundaries conditions along x directions were applied. Furthermore, we adopted a graded mesh approach to accurately model gold nanoparticles in a 2D-FDTD simulation while maintaining tractable simulation times. The fine mesh grid size is 0.5 nm in electric field monitor region, which includes gold nanoparticles and ZnO NS. In the surrounding water region, the coarse conformal mesh accuracy setting of 4 was adopted. The convergence testing was done and the result indicated that these simulations were computationally feasible.

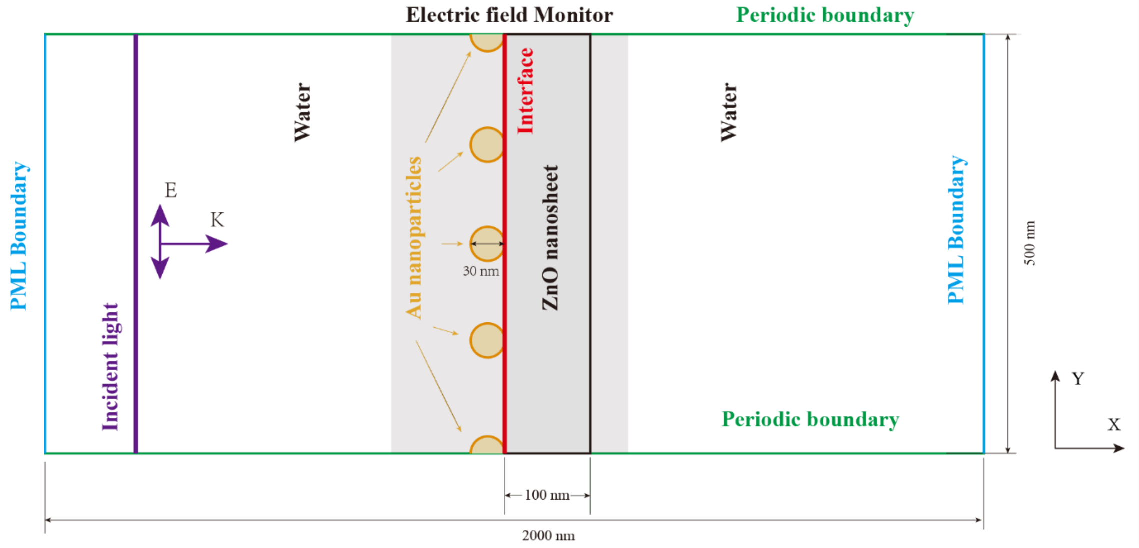

Figure S4. The schematic diagram of particle-film model and FDTD model details

According to electromagnetic theory, the intensity of light radiation is proportional to the square of electric field amplitude. Therefore, in many electromagnetic field simulations, the square of the electric field amplitude is often used to represent light irradiation intensity, which is called as local field intensity (denoted as $|E|^2$). In addition, when FDTD simulates the response to illumination of different wavelengths, it usually does not consider the impact of the incident intensity on local field. Therefore, the incident field intensity is usually normalized and set to 1. At this time, the local field intensity represents the ratio of the local field intensity to the original intensity, which is called the local field intensity enhancement factor (denoted as $|E/E_0|^2$).

Since hot electrons mainly appear at the interface between gold and zinc oxide, the mesh data at the interface between gold and zinc oxide to are averaged to obtain the average local field intensity enhancement factor (denoted as $|E/E_0|^2_{Avg}$). The calculation formula of $|E/E_0|^2_{Avg}$ is as follows:

$$|E/E_0|^2_{Avg} = 1/N^* \sum |E/E_0|^2,$$

where N denotes the numbers of FDTD mesh at the Au-ZnO interface.

Considering the relative spectral intensity $I_{relative}$ of different wavelengths in a xenon lamp, the actual average local field intensity (denoted as $|E_{actual}|^2_{Avg}$) can be obtained by multiplying the relative spectral intensity $I_{relative}$ by the average local field intensity enhancement factor $|E_{actual}|^2_{Avg}$. The calculation formula $|E_{actual}|^2_{Avg}$ is as follows:

$$|E_{actual}|^2_{Avg} = I_{relative} * |E/E_0|^2_{Avg}$$

7. Relative Electric field value along the interface considering the spectral of the irradiation source

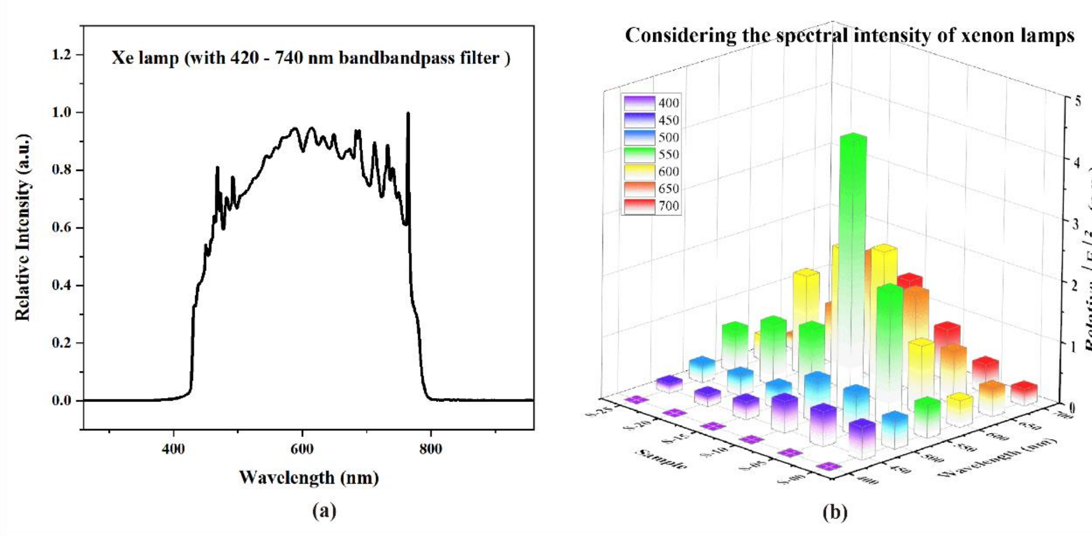

Figure S5. (a) Spectrum of xenon lamp (adding 420nm-740nm bandpass filter) (b) Actual Electric field value along the interface between ZnO NS and Au nanoparticles considering the spectral corrections of the irradiation source

Table S3. Actual electric field value along the interface with different simulated model considering the spectral corrections

Wavelength (nm)	Relative spectral intensity	S-00	S-05	S-10	S-15	S-20	S-25
400	0.00705	0.00804	0.00848	0.00765	0.00417	0.00247	0.002
450	0.54092	0.5388	0.5698	0.51794	0.29125	0.19278	0.16047
500	0.68655	0.48488	0.69831	0.69835	0.36034	0.33934	0.29732
550	0.84411	0.54137	2.17011	4.40897	1.12897	1.00307	0.69551
600	0.86643	0.43566	1.10945	2.43239	2.25638	1.61182	0.40065
650	0.90749	0.46007	0.84458	1.57897	2.00498	0.93197	0.23167
700	0.75955	0.27665	0.46274	0.82111	1.40713	0.2778	0.11956
Average actual electric field value from 400 nm -700 nm	——	0.39221	0.837639	1.495054	1.064746	0.62275	0.272454
